# Psychometric validation of the Malay CMNI-30: A study among male healthcare professionals in Malaysia

**DOI:** 10.1371/journal.pone.0320765

**Published:** 2025-04-01

**Authors:** Muhammad Iqbal Haji Mukhti, Mohd Ismail Ibrahim, Tengku Alina Tengku Ismail, Najib Majdi Yaacob

**Affiliations:** 1 Department of Community Medicine, School of Medical Sciences, Health Campus, Universiti Sains Malaysia, Kubang Kerian, Malaysia; 2 Unit of Biostatistics and Research Methodology, School of Medical Sciences, Health Campus, Universiti Sains Malaysia, Kubang Kerian, Malaysia; An-najah National University Faculty of Medicine and Health Sciences, Palestine, State Of

## Abstract

Masculinity norms significantly influence men’s health behaviors and outcomes, yet existing instruments to measure these norms often lack cultural adaptability. This study addresses this gap by validating the Malay version of the Conformity to Masculine Norms Inventory (CMNI-30) to assess masculinity norms among Malay men. A cross-sectional study was conducted with 438 male healthcare professionals in Malaysia. Confirmatory Factor Analysis (CFA) revealed that the final model, consisting of 26 items across multiple factors, demonstrated strong fit indices (χ²/df =  1.72, CFI =  0.931, TLI =  0.911, RMSEA =  0.042, SRMR =  0.052). However, certain factors, such as ‘self-reliance’ (Raykov’s rho =  0.270) and ‘violence’ (Raykov’s rho =  0.431), exhibited lower reliability, reflecting cultural nuances in their interpretation. The study concludes that the Malay CMNI-30 is a valid and culturally relevant tool for assessing masculinity norms in this context. Practical applications include using this tool to identify gender-related barriers to health-seeking behaviors and inform gender-sensitive public health interventions. Future research should validate this instrument across diverse populations to enhance its generalizability and utility.

## Introduction

Masculinity is a multifaceted construct shaped by biological, psychological, and social factors, encompassing traits, behaviors, and roles traditionally associated with men [1]. Biologically, male sex hormones significantly influence physical and psychological attributes. However, societal expectations play a crucial role in defining masculinity, emphasizing characteristics such as emotional restraint, self-reliance, stoicism, dominance, and resilience [2].

Masculinity norms profoundly impact health behaviors, often discouraging men from seeking healthcare. Norms like emotional restraint and self-reliance deter men from expressing health concerns or seeking medical assistance, as these behaviors are perceived as signs of vulnerability [3,4]. Consequently, men who strongly adhere to these norms frequently exhibit lower health literacy, delayed healthcare utilization, and poorer health outcomes [5,6].

Cultural contexts further shape the interpretation and influence of masculinity norms on health behaviors. In Western societies, hegemonic masculinity is characterized by risk-taking and dominance, which often discourage men from engaging with health services [6]. In contrast, Arab and South Asian cultures sometimes align health-seeking behaviors with fulfilling masculine responsibilities, such as providing for one’s family [7]. Similarly, in Southeast Asia, masculinity is often closely tied to roles like family provision and career success, influencing health-seeking behaviors, particularly when these roles are perceived to be at risk [8,9]. These cultural variations highlight the importance of examining masculinity within specific societal contexts.

Despite its significance, research on masculinity norms in Malaysia remains limited, and existing tools for assessing these norms have not been culturally validated. The Conformity to Masculine Norms Inventory (CMNI) is a widely used instrument to measure adherence to traditional masculine norms. Although the CMNI has been translated and validated in various languages, it has yet to be adapted for the Malay language and cultural context. Recognizing the need for culturally specific research, this study seeks to address this gap by translating and validating the CMNI-30 for use in Malaysia. A validated Malay CMNI-30 will serve as a robust tool for understanding masculinity norms and their implications for health behaviors, thereby informing culturally appropriate public health interventions.

### Literature review

#### Masculinity norms and health behaviors.

Masculinity norms, often rooted in societal expectations, significantly shape men’s health behaviors. Traditional Masculinity Ideology (TMI) emphasizes traits such as emotional control, self-reliance, and dominance, which frequently discourage men from seeking medical help or expressing vulnerabilities [10]. Research indicates that these norms can lead to delayed healthcare utilization, lower health literacy, and increased risk-taking behaviors, ultimately contributing to poorer health outcomes [3,6].

While hegemonic masculinity in Western contexts emphasizes assertiveness and independence, masculinity is interpreted differently in other cultural settings. For instance, Arab and South Asian men may align health-seeking behaviors with fulfilling familial responsibilities, which are perceived as an extension of their masculine identity [7]. Similarly, in Southeast Asia, masculinity norms often emphasize roles like family provision and career success. These roles can strongly influence health behaviors, especially when perceived as being under threat [8,9]. Such cultural variations underscore the importance of conducting context-specific research to understand how masculinity norms impact health behaviors.

#### The Conformity to Masculine Norms Inventory (CMNI).

The CMNI, developed by Mahalik et al. (2003), is a comprehensive tool designed to assess adherence to traditional masculine norms [11]. Initially comprising 94 items across 11 subscales, the CMNI evaluates various dimensions of masculinity, including emotional control, risk-taking, and self-reliance. Over time, the CMNI has been refined into shorter versions, such as the CMNI-46, CMNI-29, and CMNI-30, to reduce participant burden while maintaining psychometric robustness [12–14]. Among these, the CMNI-30 has demonstrated strong psychometric properties, making it a preferred choice for research and clinical applications.

However, studies suggest that certain subscales may exhibit lower reliability for men of color compared to White men, highlighting the need for ongoing refinement and cultural validation [15].

#### Cross-cultural adaptations of the CMNI.

The CMNI has been translated and validated in various cultural contexts, including Chinese, Spanish, Russian, and German [16–19]. These adaptations demonstrate the tool’s global applicability but also reveal challenges in maintaining reliability and validity across diverse populations. For example, cultural differences in interpreting constructs like dominance or self-reliance can influence the tool’s effectiveness. Addressing these differences through cultural adaptation and validation is essential to ensure the CMNI remains relevant and accurate in diverse settings.

#### Research gap.

Despite its widespread use, the CMNI-30 has not been validated in Malay, limiting its applicability in Malaysia. Linguistic and cultural differences can significantly affect how masculinity norms are perceived and assessed, necessitating a culturally tailored tool. Validating the CMNI-30 in Malay will enable researchers to explore the relationship between masculinity norms and health behaviors in this context, offering valuable insights for designing effective public health intervention

## Methods

### Study design

This study employed a cross-sectional design to validate the Malay version of the Conformity to Masculine Norms Inventory (CMNI-30). Cross-sectional studies are widely used in psychometric validation as they provide a snapshot of the relationships between variables and allow for the evaluation of the instrument’s reliability and validity in a specific population [20,21]. Data collection was conducted from 20/05/2023 to 31/07/20 focusing on male healthcare professionals from diverse clinical settings in Malaysia. This design was chosen to ensure the efficient collection of data within a single time frame, minimizing potential confounding effects due to longitudinal changes.

### Study settings

The study was conducted at Hospital Pakar Universiti Sains Malaysia, Kubang Kerian, Kelantan, Malaysia. Male healthcare workers aged 18 to 60 years were eligible to participate. Non-Malaysian citizens and those unavailable during the study period were excluded.

### Sample size determination and sampling method

The required sample size was calculated following factor analysis guidelines, which recommend a minimum of 10 participants per indicator variable [22,23]. To ensure sufficient statistical power, at least 300 participants were deemed necessary [24,25]. A 20% margin was added to account for potential dropout or incomplete responses [26], resulting in a target sample of 360 participants.

A total of 455 participants were recruited using simple random sampling to maximize representativeness. After excluding 17 incomplete responses, 438 participants were included in the final analysis.

### Instrument

The CMNI-30 is a self-administered, 30-item questionnaire rated on a six-point Likert scale (1 =  “strongly disagree” to 6 =  “strongly agree”) [14]. It measures ten specific masculine norms, each represented by three items: emotional control, winning, playboy, violence, heterosexual self-preservation, pursuit of status, primacy of work, power over women, self-reliance, and risk-taking [18]. Nine items are reverse-coded. Subscale scores, calculated as the average of relevant items, provide more detailed insights than total scores, indicating higher conformity to these norms. The CMNI-30 is specifically designed to measure particular masculine norms rather than general constructs [14]. See S1 Appendix for additional details.

### Translation and validation process

Accurate and consistent translation of questionnaires is crucial, with international standards providing essential guidance [27]. Following recent translation frameworks, a seven-stage process was undertaken [27,28]. Initially, two native Malay translators; one from a medical background and the other from a non-medical background, independently translated the questionnaire. These translations were reconciled into a single document after addressing discrepancies through discussions with translators, study team members, and a linguistic expert.

The next step involved backward translation by two additional independent translators, also from medical and non-medical backgrounds. Harmonization followed, where all translated reports were reviewed and compared with the original English version. Discrepancies and challenging terms were identified and revised, resulting in the preliminary Malay version titled “Kepatuhan kepada Norma Maskulin”, designed to measure adherence to masculine norms. The translated version sample is provided in the supplementary document. The pre-survey assessment of the Malay version of the CMNI-30 included validation processes to ensure the instrument’s accuracy. Expert panels evaluated content validity by assessing the relevance of items to the targeted construct. Six experts specializing in public health and family medicine, with a focus on men’s health, rated the relevance of all 30 items using a 4-point scale [29,30]. Additionally, face validation was conducted through cognitive debriefing with 10 public servant respondents, ensuring the clarity and comprehensibility of the translated items [29,31].

### Data analysis

Data entry was performed in Microsoft Excel, with a custom calculator used for validity analysis. Confirmatory Factor Analysis (CFA) and reliability assessments were conducted in RStudio using the lavaan package [25] and semTools [32]. Mplus 8.3 was used to generate the CFA path diagram [33].

### Ethical statement

This study received ethical approval from the USM Human Research Ethics Committee on 16th May 2024 (USM/JEPeM/KK/23010115). To ensure the ethical protection of all participants, the research team provided a thorough explanation of the study’s purpose, procedures, and confidentiality measures. Participants were informed that their involvement was entirely voluntary and that they could withdraw from the study at any time without any negative consequences. Written consent was taken from those who voluntarily agreed to participate, after which they were asked to sign the consent form, and the questionnaire was administered. All collected data were anonymized to ensure participant confidentiality and were securely stored in a locked location accessible only to the research team.

## Results

### Descriptive analysis

S1 Table summarizes the demographic characteristics of 438 male participants, with an average age of 35.2 years (SD ± 7.38). Almost all participants were Muslim (99.8%) and Malay (99.3%). Most participants had diploma-level education (44.1%) or upper secondary education (37.0%). Common occupations were attendants (57.1%) and nurses (35.4%). The average length of service was 10.3 years (SD ± 7.80), with over half having less than 10 years of experience. More than 80% were married, with 38.6% having 1 to 2 children. Monthly income was predominantly below RM2500 (47%) or between RM2500-RM5000 (42.9%).

### Confirmatory Factor Analysis (CFA)

The Confirmatory Factor Analysis (CFA) process involved nine models. Model 9 was selected as the best-fitting model after iterative adjustments, including the removal of items with low factor loadings and the correlation of errors with high modification indices (MI). The final model retained 26 items, with factor loadings ranging from 0.324 to 0.952. Model 9 demonstrated strong fit indices, including χ²/df =  1.72, CFI =  0.931, TLI =  0.911, RMSEA =  0.042, and SRMR =  0.052.

Although discriminant validity was partially achieved, certain factors (e.g., F2: 0.556, F9: 0.438) exhibited multicollinearity with other factors. Convergent validity was acceptable for four factors; however, six factors—such as winning, violence, and self-reliance—did not meet the Average Variance Extracted (AVE) threshold. A detailed description of the model is as follows:

#### Model 1: Initial model.

The initial model included all 30 items from the CMNI-30; however, it exhibited a poor fit due to two items (X12 and X27) having very low factor loadings (0.016 and 0.183, respectively), both falling below the acceptable threshold of 0.30. To improve the overall model fit, these items were removed in subsequent iterations.

#### Model 2 to Model 4: Refinement through item removal.

In Model 2, items X12 and X27 were excluded, leading to an improvement in factor loadings. However, the overall model fit indices remained below the desired thresholds. To further enhance the model, error terms between item pairs with high modification indices (MI) were correlated in Model 3. This adjustment resulted in some improvement but was still insufficient for an acceptable fit. In Model 4, another item (X17) was removed due to its factor loading of 0.399, which, although higher than the previously excluded items, was still below the acceptable threshold.

#### Model 5 and Model 6: Item removal for better fit.

In Model 5, further efforts were undertaken to improve the model by correlating additional item errors with high modification indices (MI). Despite these adjustments, Model 5 still did not achieve acceptable fit standards. Consequently, items with relatively low factor loadings (X1, X4, and X5) were identified for potential removal. Model 6 was then developed by removing item X1, which resulted in a further improvement in the model fit.

#### Model 7 to Model 9: Final model selection.

Models 7 and 8 involved further adjustments by correlating errors and refining the item set. Model 9 was subsequently developed by removing item X5 and correlating additional errors with high modification indices (MI) identified in Model 8. These adjustments resulted in the final model, which retained 26 items.

#### Convergent and discriminant validity.

While most factors demonstrated acceptable convergent and discriminant validity, certain issues persisted. Convergent validity, which evaluates whether items intended to measure the same construct are indeed related, was acceptable for four factors. However, six factors—winning, violence, heterosexual self-preservation, pursuit of status, power over women, and self-reliance—did not meet the recommended threshold for Average Variance Extracted (AVE), with values below 0.50.

Discriminant validity, which ensures that factors measuring different constructs are distinct, was partially achieved. For most factors, the square roots of the AVE values exceeded their correlations with other factors, indicating sufficient discriminant validity. However, factors such as F2 (winning) and F9 (self-reliance) exhibited multicollinearity with other factors (e.g., F4 and F5), suggesting potential overlap between these constructs.

In summary, Model 9 retained 26 items, demonstrated a good fit across multiple indices, and provided a robust structure for the Malay version of the CMNI-30. Nevertheless, further refinement may be necessary to address issues with convergent validity, particularly in factors such as self-reliance and violence. The findings are detailed in S2 and S3 Tables.

### Reliability analysis

Reliability, assessed using Raykov’s rho, ranged from 0.270 to 0.807. Factors such as primacy of work (ρ =  0.807) and playboy (ρ =  0.774) demonstrated strong internal consistency, while self-reliance (ρ =  0.270), violence (ρ =  0.431), and winning (ρ =  0.475) exhibited lower reliability scores. The Corrected Item-Total Correlations (CI-TC) were generally within acceptable limits; however, items X25 and X26 in the self-reliance factor had CI-TC values of 0.171, indicating a weaker contribution to the overall construct. Despite this, the inter-item correlations for most factors were within the acceptable range (see S4 and S5 Tables). Although the self-reliance factor showed lower composite reliability, its inter-item correlation of 0.171 met the minimum threshold for consistency.

#### Path diagram of Model 9: Final structure of the CMNI-30 in the Malay context.

**Fig 1:**
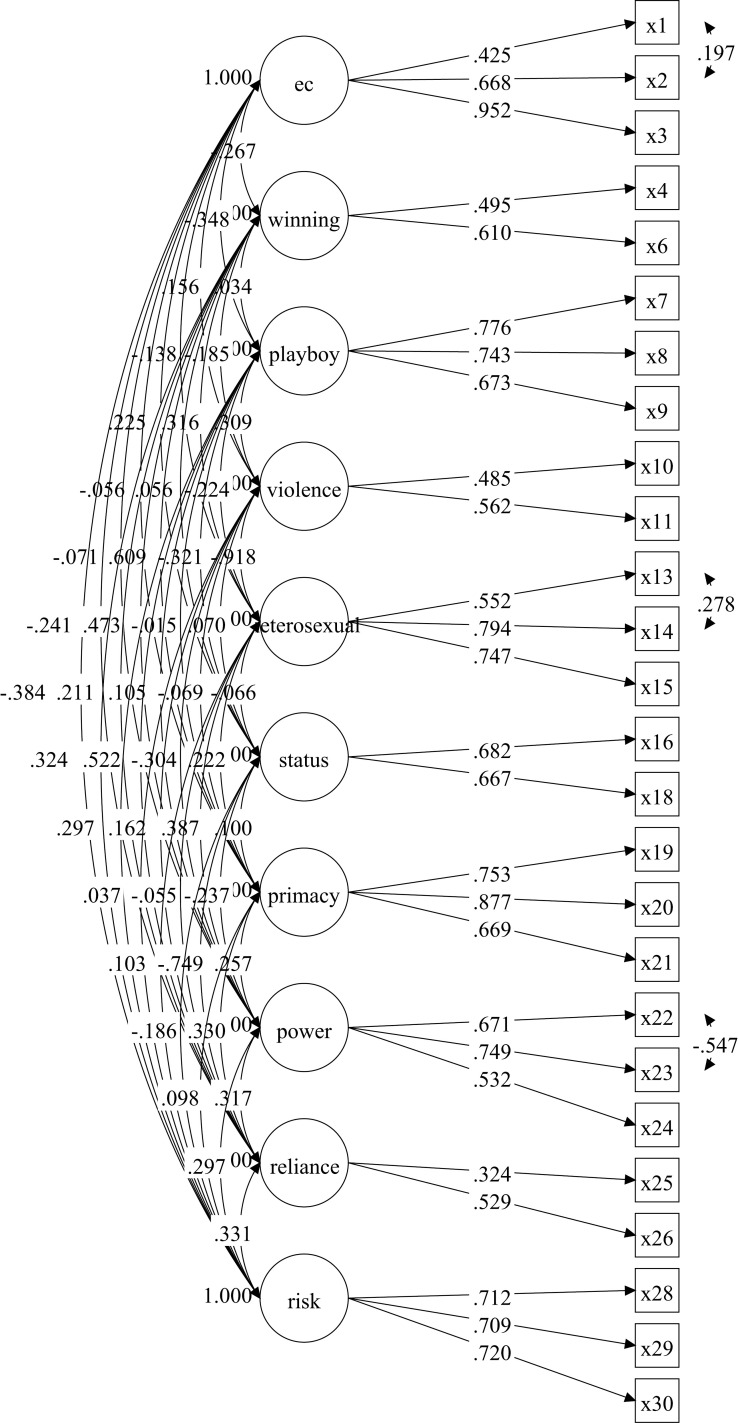
The path diagram of Model 9.

Fig 1 shows the path diagram for Model 9, illustrating the relationships between the ten latent variables (factors) of the CMNI-30 and their respective observed items. Each arrow represents a factor loading, indicating the strength of the connection between the items and their underlying constructs, with loadings ranging from 0.324 to 0.952. The latent variables are shown as circles, while the items are displayed as rectangles. Error terms, depicted by small circles attached to each item, represent unexplained variance, and several error terms are correlated (shown as curved lines) to improve the model’s fit based on high modification indices. This diagram visually summarizes the final structure of the CMNI-30 after adjustments, highlighting the relationships and dependencies between the items and their factors in the Malay context

## Discussion

The CFA of the Malay version of the CMNI-30 revealed that the removal of certain items was necessary to improve the model’s fitness and psychometric properties [14,15]. These adjustments enhanced the construct validity by refining the model fit indices. However, concerns arose about whether reducing certain factors to two items, from the original three, could still effectively measure the intended constructs. Conventionally, three to four items per factor are preferred to ensure validity [34]. A higher number of items is generally advantageous because with only two items, construct validity can be compromised if one item is misunderstood or poorly worded [35]. Although two-item constructs can be viable if they demonstrate reliability and meet model-fit criteria [36,37], this study revealed significant issues with discriminant and convergent validity, particularly in factors reduced to two items.

Factors with more items tend to better capture the construct and improve validity and reliability [35]. However, cultural interpretations of certain constructs may also contribute to validity issues. For instance, the ‘self-reliance’ factor, which reflects the traditional masculine ideal of independence, may conflict with collectivist values prevalent in Malay culture, where interdependence and communal support are highly valued [38]. Similarly, the ‘violence’ factor may be viewed as less relevant or acceptable in a cultural context that emphasizes harmony and non-confrontation as societal ideals [39]. These cultural nuances may lead to weaker convergent and discriminant validity for these factors.

Masculinity norms, including those measured by the CMNI-30, vary widely across cultural and religious contexts. In the Malay cultural setting, influenced significantly by Islamic teachings, some masculine norms may align closely with local values, while others may diverge. For example, the ‘playboy’ norm, which emphasizes promiscuity and casual relationships, contrasts sharply with Islamic principles that prioritize modesty and fidelity [40]. Respondents’ discomfort with this construct may affect its validity in this cultural context. Similarly, the ‘power over women’ norm, while reflective of traditional patriarchal structures, may conflict with the increasing emphasis on gender equity and shared decision-making in Malay Muslim households [41].

On the other hand, certain norms, such as ‘emotional control’ and ‘risk-taking,’ may resonate with Malay masculinity as they align with societal expectations for men to demonstrate resilience and courage [42]. These alignments and divergences underscore the importance of adapting items to ensure cultural sensitivity while retaining the theoretical integrity of the CMNI-30.

A cross-cultural comparison highlights these nuances. For instance, studies in Western contexts often report high conformity to the ‘playboy’ norm as a marker of masculinity, whereas in Southeast Asia, this norm is less socially acceptable [14,16]. Similarly, while the ‘power over women’ norm has been critiqued in Western feminism for perpetuating inequality, its relevance in Malaysia may differ, as societal expectations often emphasize a balance of roles within a religious framework [41]. Including these cultural considerations strengthens the CMNI-30’s ability to capture the complexity of masculinity in the Malay context.

Future studies should explore these cultural nuances in greater depth, using qualitative approaches to understand how Malay men perceive and internalize these norms. Additionally, cross-validation studies with other Southeast Asian populations could further refine the instrument, ensuring its relevance across diverse cultural settings.

The low composite reliability of the ‘self-reliance’ factor (Raykov’s rho =  0.270) highlights the need for refinement. This poor performance could be due to a mismatch between the factor’s traditional framing and the collectivist cultural values of Malay society. In this context, self-reliance may be understood less as individual independence and more as the ability to function autonomously within a communal or group framework [38]. Additionally, the phrasing of items may not have adequately captured the local interpretation of self-reliance, leading to inconsistencies in how respondents interpreted and rated these items.

To address these issues, future iterations of the Malay CMNI-30 could consider refining these factors by adding culturally relevant items or adapting existing items to align more closely with Malay cultural norms. For example, the ‘self-reliance’ factor could include items emphasizing self-efficacy within a community context [43], and the ‘violence’ factor could explore non-physical forms of dominance or assertiveness that resonate with local values [44]. Pilot testing these modifications in diverse Malay-speaking populations would help ensure the constructs are both valid and culturally appropriate.

The CFA process involved several iterative model adjustments aimed at improving model fit and construct validity [45,46]. Model 9 was selected as the best-fitting model after making a series of theoretical and statistical modifications. These included the removal of items with low factor loadings and correlating error terms with high modification indices (MI) [35,47]. Each of these steps was guided by both theoretical considerations and statistical results, ensuring that the final model accurately represented the intended constructs of masculinity norms.

For the removal of items, specific items with low factor loadings were excluded because they did not strongly correlate with the latent constructs they were meant to measure. This was particularly true for items within the ‘self-reliance’ and ‘violence’ factors. The removal of these items helped streamline the model and improved the overall fit by focusing on items that more clearly reflected the theoretical dimensions of these constructs [48]. For instance, the ‘self-reliance’ factor, which is culturally nuanced, may have included items that did not resonate with the Malay context, leading to low loadings. Removing these items ensured that the factor better reflected Malay masculine norms of self-efficacy within a group context.

Additionally, correlating error terms was based on modification indices that suggested a high degree of shared variance between certain items, which could be attributed to conceptual overlap rather than measurement error [21]. For example, items related to ‘winning’ and ‘status’ were correlated to better account for the shared thematic content between them. While error correlations can be problematic if overused, in this case, they were deemed necessary to improve model fit and align the factor structure with the theoretical conceptualization of masculinity in the Malay context.

The final model included 26 items, with factor loadings ranging from 0.324 to 0.952, and demonstrated strong fit indices (χ²/df =  1.72, CFI =  0.931, TLI =  0.911, RMSEA =  0.042, SRMR =  0.052) [49,50]. This refined model better captures the key dimensions of masculinity norms while maintaining both statistical robustness and theoretical integrity

Measurement errors are a critical concern in psychometric assessments. Errors can result from random factors such as respondent fatigue, as well as systematic factors like biased item wording and cultural differences [20]. Although the Malay version of the CMNI-30 underwent careful translation and pre-testing, measurement errors can still obscure true relationships between items and compromise validity. Clarity and precision in item wording are crucial to avoid these errors [27]. Another factor that may have influenced the reliability and validity of the results is the 6-point Likert scale used in this study. Too many or too few response options can lead to respondent fatigue or reduced variability, both of which can negatively impact data quality [51].

The sample size is another significant factor in the reliability of the findings. A larger sample size provides greater statistical power and more stable parameter estimates, which is crucial for questionnaire-based research [52]. Although this study’s sample size of 438 was adequate, future studies could benefit from larger samples, as suggested by Comrey and Lee (1992), who recommend at least 500 participants to ensure robust factor analysis results. The relatively small sample size in this study may have contributed to the observed validity and reliability issues, particularly in the underperforming factors [52].

Similar issues with validity and reliability have been observed in studies of the CMNI-30 in other populations. Rochelle and Yim (2015) found that although the 10-factor structure was supported in the Hong Kong context, some items failed to meet the factor loading cutoff. Similarly, Komlenac et al. (2023) confirmed the 10-factor structure of the German CMNI-30 but noted partial issues with discriminant validity [19]. In the Russian version of the CMNI-30, Krivoshchekov et al. (2022) also reported lower internal consistency for certain subscales [18]. These findings underscore the importance of continuous refinement and validation of the CMNI-30 across diverse cultural settings to ensure it accurately measures masculine norms.

It is also worth noting that healthcare professionals may differ significantly from the general population in their conformity to traditional masculine norms. Research indicates that male healthcare workers often navigate a balance between professional expectations of empathy and societal masculine norms, potentially leading to less adherence to traditional masculinity ideals [53]. Their professional training, which emphasizes empathy and patient-centered care, may counteract some aspects of traditional masculinity, such as emotional control or self-reliance [54]. These differences underscore the need to validate the Malay CMNI-30 among other male groups to ensure its applicability in various cultural and socio-economic contexts.

### Practical application

The validated Malay CMNI-30 represents a significant advancement in understanding masculinity norms within the Malaysian cultural context. This instrument has practical applications in both clinical and public health settings. For example, it can serve as a valuable tool for identifying masculinity-related barriers to health-seeking behaviors, such as emotional control or self-reliance, which may discourage men from seeking timely medical care or engaging in preventive health practices. By identifying these barriers, healthcare providers can develop gender-sensitive interventions that address these norms and encourage more proactive health behaviors among men.

In public health settings, the Malay CMNI-30 could be employed in community-based programs aimed at promoting men’s health. For instance, the tool could guide the design of campaigns targeting risk-taking behaviors, emphasizing healthier alternatives that align with positive masculinity traits such as resilience and leadership. Additionally, it can support efforts to address mental health stigma among men by identifying norms that discourage emotional expression and fostering a more supportive environment for mental health discussions.

Furthermore, the instrument’s adaptability to other Southeast Asian contexts makes it a valuable resource for cross-cultural research and regional collaborations. By using the Malay CMNI-30, researcher and policymakers can better understand the role of masculinity norms in shaping health behaviors and outcomes, enabling the design of culturally tailored, evidence-based health interventions. Future studies could build on this work by applying the instrument to diverse populations, including rural and non-healthcare professional groups, to further validate its utility and generalizability.

### Conclusion

This study validated the Malay version of the Conformity to Masculine Norms Inventory (CMNI-30) for use among male healthcare professionals in Malaysia. The results confirmed that the Malay CMNI-30 is a psychometrically sound and culturally relevant instrument, with acceptable model fit indices and reliability across most factors. However, certain factors, such as ‘self-reliance’ and ‘violence,’ exhibited lower reliability, highlighting the influence of cultural nuances on the interpretation of these constructs.

The findings underscore the importance of culturally adapting and validating psychological instruments to ensure their accuracy and applicability in specific populations. The Malay CMNI-30 provides a valuable tool for exploring the relationship between masculinity norms and health behaviors in Malaysia, enabling gender-sensitive health interventions and research. By identifying masculine norms that may act as barriers to health-seeking behaviors, this instrument can inform the development of targeted public health strategies aimed at improving men’s health outcomes.

Future research should validate the Malay CMNI-30 in more diverse populations, including rural communities and non-healthcare professionals, to enhance its generalizability. Further refinement of underperforming factors, such as ‘self-reliance,’ may also improve the instrument’s reliability and cultural alignment. Overall, the Malay CMNI-30 represents a significant contribution to the field, advancing our understanding of masculinity and its impact on health in Southeast Asia.

### Limitations and recommendations

This study has several limitations. The sample, drawn from male healthcare professionals in Kelantan, Malaysia, is likely to exhibit unique adherence to masculine norms influenced by their exposure to healthcare-related training and work environments. This homogeneity may limit the generalizability of the findings to the broader Malay male population, particularly those in non-professional or rural settings. Healthcare professionals may have more progressive attitudes toward gender roles and health behaviors compared to other groups. Future studies should focus on diverse demographic groups, including non-professional and rural populations, to validate the tool’s applicability and ensure wider generalizability. Additionally, the cross-sectional design limits the ability to infer causality. Further studies with larger and more diverse samples across different cultural and socio-economic groups are necessary to validate the Malay version of the CMNI-30. Cross-validation in independent cohorts and using a broader sample, as suggested by Hair et al. (2010), would provide more precise parameter estimates and enhance the generalizability of the findings [55].

Moreover, it is recommended to assess the psychometric properties of other versions of the CMNI, such as the CMNI-94 and CMNI-46, in addition to the CMNI-30. This broader approach may improve convergent and discriminant validity, as well as reliability [18]. To ensure data integrity, future studies should implement stringent data collection and quality control protocols, such as standardized administration procedures and post-collection data validation [34]. Advanced statistical methods, such as Exploratory Factor Analysis (EFA), should also be considered to explore underlying factor structures more deeply [56]. Incorporating qualitative feedback from respondents could further enhance the relevance and clarity of the items, ensuring that the CMNI-30 is culturally sensitive and free from misinterpretation [27].

Future research should aim to assess the psychometric properties of the Malay CMNI-30 in more diverse populations, including those with lower educational attainment, individuals in rural settings, and those engaged in non-healthcare professions. Such efforts would enhance the tool’s applicability across a broader spectrum of the Malay male population and provide a more comprehensive understanding of masculinity norms in Malaysia.

Future research should focus on revising the ‘self-reliance’ and ‘violence’ scales to incorporate items that better reflect Malay cultural norms. Collaborative workshops with cultural experts and focus groups with male participants from diverse socio-economic backgrounds could provide valuable insights into culturally relevant constructs [20]. Furthermore, conducting exploratory and confirmatory factor analyses on these revised scales would help establish their psychometric robustness in this context.

## Supporting information

S1 Appendix Malay version of CMNI-30
(DOCX)

S1 Table Sociodemographic characteristic
(DOCX)

S2 Table Model fit indices
(DOCX)

S3 Table Inter-factor correlations
(DOCX)

S4 Table Average Variance Extracted
(DOCX)

S5 Table Factor loading Model 9 items
(DOCX)
