# Supplementary material for: Psychometric validation of the Malay CMNI-30: A study among male healthcare professionals in Malaysia
Source: PLoS One. 2025 Apr 1;20(4):e0320765. doi: 10.1371/journal.pone.0320765 (PMC11960922; doi:10.1371/journal.pone.0320765)
Supplement: S1 Appendix — (DOCX) [file pone.0320765.s001.docx]

**SUPPLEMENTARY DOCUMENT**

S1 Appendix. Malay version of CMNI-30

| **Soal Selidik Inventori Kepatuhan kepada Norma Maskulin (CMNI-30)** | | | | | | | |
| --- | --- | --- | --- | --- | --- | --- | --- |
| Sila lengkapkan soal selidik dengan membulatkan nombor yang menunjukkan tahap setuju atau tidak setuju anda dengan setiap pernyataan. Berikan satu jawapan sahaja bagi setiap pernyataan. | | **Sangat tidak setuju** | **Tidak setuju** | **Agak Tidak Setuju** | **Agak**  **setuju** | **Setuju** | **Sangat setuju** |
|  | Saya cenderung untuk berkongsi perasaan saya | 1 | 2 | 3 | 4 | 5 | 6 |
|  | Saya akan menjadi marah jika orang beranggapan saya suka sesama jantina | 1 | 2 | 3 | 4 | 5 | 6 |
|  | Saya tidak suka dengan sebarang bentuk keganasan | 1 | 2 | 3 | 4 | 5 | 6 |
|  | Saya rasa menyusahkan apabila saya perlu meminta bantuan | 1 | 2 | 3 | 4 | 5 | 6 |
|  | Saya suka meluahkan perasaan saya ketika bercakap dengan orang lain | 1 | 2 | 3 | 4 | 5 | 6 |
|  | Kerja adalah keutamaan bagi saya | 1 | 2 | 3 | 4 | 5 | 6 |
|  | Perasaan paling gembira di dunia adalah apabila saya menang sesuatu | 1 | 2 | 3 | 4 | 5 | 6 |
|  | Saya seronok apabila mengambil tindakan berisiko | 1 | 2 | 3 | 4 | 5 | 6 |
|  | Saya berpendapat bahawa menjadi seseorang yang penting adalah membuang masa | 1 | 2 | 3 | 4 | 5 | 6 |
|  | Wanita dalam hidup saya perlu mengikut perintah saya | 1 | 2 | 3 | 4 | 5 | 6 |
|  | Saya akan sangat marah jika seseorang beranggapan saya suka sesama jantina | 1 | 2 | 3 | 4 | 5 | 6 |
|  | Saya akan sering menukar pasangan intim saya sekiranya saya boleh | 1 | 2 | 3 | 4 | 5 | 6 |
|  | Saya suka bercakap mengenai perasaan saya | 1 | 2 | 3 | 4 | 5 | 6 |
|  | Saya mendapati sesuatu yang menyeronokkan untuk berpacaran dengan lebih daripada seorang pada satu masa | 1 | 2 | 3 | 4 | 5 | 6 |
|  | Ia sama sekali tidak pernah baik bagi saya jika saya menjadi seorang yang ganas | 1 | 2 | 3 | 4 | 5 | 6 |
|  | Ianya menjengkelkan jika ada orang mengandaikan yang saya suka sesama jantina | 1 | 2 | 3 | 4 | 5 | 6 |
|  | Saya meletakkan diri saya dalam situasi yang berisiko | 1 | 2 | 3 | 4 | 5 | 6 |
|  | Keadaan menjadi lebih baik jika lelaki yang memimpin | 1 | 2 | 3 | 4 | 5 | 6 |
|  | Saya rasa seronok apabila kerja menjadi keutamaan saya | 1 | 2 | 3 | 4 | 5 | 6 |
|  | Saya benci jika menjadi orang penting | 1 | 2 | 3 | 4 | 5 | 6 |
|  | Saya akan melakukan apa sahaja untuk mencapai sesuatu kemenangan | 1 | 2 | 3 | 4 | 5 | 6 |
|  | Saya tidak pernah meminta pertolongan | 1 | 2 | 3 | 4 | 5 | 6 |
|  | Saya perlu mengutamakan kerja saya berbanding perkara lain | 1 | 2 | 3 | 4 | 5 | 6 |
|  | Saya suka apabila lelaki berkuasa ke atas wanita | 1 | 2 | 3 | 4 | 5 | 6 |
|  | Saya berasa senang jika saya mempunyai ramai pasangan intim | 1 | 2 | 3 | 4 | 5 | 6 |
|  | Saya ambil risiko | 1 | 2 | 3 | 4 | 5 | 6 |
