# Supplementary material for: Psychometric validation of the Malay CMNI-30: A study among male healthcare professionals in Malaysia
Source: PLoS One. 2025 Apr 1;20(4):e0320765. doi: 10.1371/journal.pone.0320765 (PMC11960922; doi:10.1371/journal.pone.0320765)
Supplement: S1 Table — (DOCX) [file pone.0320765.s002.docx]

**SUPPLEMENTARY DOCUMENT**

S1 Table. Sociodemographic characteristics of male respondents (n= 438).

| **Characteristic** | | | **Mean (SD)** | ***n* (%)** |
| --- | --- | --- | --- | --- |
| Age (Years) | | | 35.2 (7.38) |  |
| Religion | | |  |  |
|  | Islam | |  | 437 (99.8) |
|  | Christian | |  | 1 (0.2) |
| Ethnicity | | |  |  |
|  | Malay | |  | 435 (99.3) |
|  | Iban | |  | 1 (0.2) |
|  | Others | |  | 2 (0.5) |
| Educational level | | |  |  |
|  | Lower secondary | |  | 18 (4.1) |
|  | Upper secondary | |  | 162 (37.0) |
|  | Malaysia skills certificate | |  | 29 (6.6) |
|  | Diploma | |  | 193 (44.1) |
|  | Bachelor’s degree | |  | 24 (5.5) |
|  | Master’s degree | |  | 12 (2.7) |
| Occupation | | |  |  |
|  | Attendant | |  | 250 (57.1) |
|  | Nurse | |  | 155 (35.4) |
|  | Assistant Medical Officer | |  | 15 (3.4) |
|  | Medical Officer | |  | 14 (3.2) |
|  | Operational assistant | |  | 3 (0.7) |
|  | Public assistant | |  | 1 (0.2) |
| Length of service | | | 10.3 (7.80) |  |
|  | <10 years | |  | 237 (54.1) |
|  | 10 – 19 years | |  | 163 (37.2) |
|  | 20 – 29 years | |  | 18 (4.1) |
|  | 30 – 39 years | |  | 20 (4.6) |
| Marital status | | |  |  |
|  | Married | |  | 365 (83.3) |
|  | Single | |  | 66 (15.1) |
|  | Divorce/widower | |  | 7 (1.6) |
| Number of children | | | 2.0 (1.70) |  |
|  | None | |  | 106 (24.2) |
|  | 1 – 2 | |  | 169 (38.6) |
|  | 3 – 4 | |  | 132 (30.1) |
|  | 5 – 6 | |  | 24 (5.5) |
|  | ≥ 7 | |  | 7 (1.6) |
| Monthly individual income (RM) | | |  |  |
|  | | < 2500 |  | 206 (47.0) |
|  | | 2500 – 5000 |  | 188 (42.9) |
|  | | 5001 – 7000 |  | 31 (7.1) |
|  | | 7001 – 11,000 |  | 13 (3.0) |
