# Supplementary material for: Psychometric validation of the Malay CMNI-30: A study among male healthcare professionals in Malaysia
Source: PLoS One. 2025 Apr 1;20(4):e0320765. doi: 10.1371/journal.pone.0320765 (PMC11960922; doi:10.1371/journal.pone.0320765)
Supplement: S2 Table — (DOCX) [file pone.0320765.s003.docx]

**SUPPLEMENTARY DOCUMENT**

S2 Table. Model fit indices with respective least factor loadings and error of item-to-item with high modification indices following CFA of the Malay version of the CMNI-30 Questionnaire.

| **Model** | **χ2 (df)** | **Normed chi-square (χ2/df)** | **SRMR** | **RMSEA (90% CI)** | **CFI** | **TLI** | **AIC** | **BIC** |
| --- | --- | --- | --- | --- | --- | --- | --- | --- |
| 1 | 760.0 (360) | 2.11* | 0.067 | 0.053 (0.048, 0.058) | 0.862 | 0.833 | 39,714.4 | 40,143.0 |
| 2 | 592.4 (305) | 1.94* | 0.057 | 0.049 (0.043, 0.054) | 0.896 | 0.872 | 36,844.3 | 37,256.6 |
| 3 | 553.3 (301) | 1.84* | 0.056 | 0.046 (0.040, 0.052) | 0.909 | 0.886 | 36,810.9 | 37,239.5 |
| 4 | 537.2 (279) | 1.93* | 0.056 | 0.048 (0.042, 0.054) | 0.905 | 0.880 | 35,342.3 | 35,746.4 |
| 5 | 502.4 (276) | 1.82* | 0.055 | 0.045 (0.039, 0.052) | 0.916 | 0.894 | 35,310.7 | 35,727.1 |
| 6 | 491.4 (254) | 1.93* | 0.055 | 0.048 (0.042, 0.055) | 0.909 | 0.884 | 33,957.9 | 34,353.9 |
| 7 | 461.5 (251) | 1.84* | 0.054 | 0.046 (0.039, 0.052) | 0.920 | 0.896 | 33,930.2 | 34,338.4 |
| 8 | 466.3 (254) | 1.84* | 0.052 | 0.046 (0.039, 0.052) | 0.919 | 0.897 | 33,933.3 | 34,329.2 |
| 9 | 431.3 (251) | 1.72* | 0.052 | 0.042 (0.036, 0.049) | 0.931 | 0.911 | 33,901.8 | 34,310.0 |

Model 1: Lowest FL (X12 = 0.016, X27 = 0.183). Model 2: MI (X1~X2 = 7.210, X1~X3 = 4.637, X13~X14 = 8.835, X13~X15 = 5.574, X16~X17 = 4.119, X16~X18 = 10.171, X22~X23 = 15.916, X22~X24 = 8.283). Model 3: Lowest FL (X25 = 0.341, X17 = 0.399). Model 4: MI (X1~X2 = 7.752, X1~X3 = 4.675, X13~X14 = 8.731, X13~X15 = 5.650, X22~X23 = 16.386, X22~X24 = 8.161). Model 5: Lowest FL (X25 = 0.324, X1 = 0.423, X4 = 0.435, X5 = 0.463). Model 6: MI (X4~X6 = 4.202, X13~X14 = 9.232, X13~X15 = 5.633, X22~X23 = 16.477, X22~X24 = 7.790). Model 7: Lowest FL (X25 = 0.327, X4 = 0.362). Model 8: MI (X1~X2 = 7.241, X1~X3 = 4.496, X13~X14 = 7.663, X13~X15 = 5.494, X22~X23 = 17.368, X22~X24 = 9.605). Model 9: Lowest FL X25 = 0.324. Indicator: * = p-value <0.001. Abbreviations: CFA = confirmatory factor analysis; χ2 = chi-square; df = degree of freedom; SRMR = standardized root mean square residual; RMSEA = root mean square error of approximation; CI = confidence interval; CFI = comparative fit index; TLI = Tucker-Lewis fit index; AIC = Akaike information criterion; BIC = Bayesian information criterion; FL = factor loading; MI = modification index.
