# Supplementary material for: Psychometric validation of the Malay CMNI-30: A study among male healthcare professionals in Malaysia
Source: PLoS One. 2025 Apr 1;20(4):e0320765. doi: 10.1371/journal.pone.0320765 (PMC11960922; doi:10.1371/journal.pone.0320765)
Supplement: S3 Table — (DOCX) [file pone.0320765.s004.docx]

**SUPPLEMENTARY DOCUMENT**

S3 Table. Inter-factor correlations and the square root of Average Variance Extracted (AVE) of all ten factors.

| **Factor** | **F1** | **F2** | **F3** | **F4** | **F5** | **F6** | **F7** | **F8** | **F9** | **F10** |
| --- | --- | --- | --- | --- | --- | --- | --- | --- | --- | --- |
| **F1** | **0.715** |  |  |  |  |  |  |  |  |  |
| **F2** | -0.267 | **0.556** |  |  |  |  |  |  |  |  |
| **F3** | -0.348 | 0.034 | **0.732** |  |  |  |  |  |  |  |
| **F4** | 0.156 | -0.185 | 0.309 | **0.525** |  |  |  |  |  |  |
| **F5** | -0.138 | 0.316 | -0.224 | -0.918 | **0.706** |  |  |  |  |  |
| **F6** | 0.225 | 0.056 | -0.321 | 0.070 | -0.066 | **0.675** |  |  |  |  |
| **F7** | -0.056 | 0.609 | -0.015 | -0.069 | 0.222 | 0.100 | **0.771** |  |  |  |
| **F8** | -0.071 | 0.473 | 0.105 | -0.304 | 0.387 | -0.237 | 0.257 | **0.657** |  |  |
| **F9** | -0.241 | 0.211 | 0.522 | 0.162 | -0.055 | -0.749 | 0.330 | 0.317 | **0.438** |  |
| **F10** | -0.384 | 0.324 | 0.297 | 0.037 | 0.103 | -0.186 | 0.098 | 0.297 | 0.330 | **0.713** |

**F1** = Emotional control; **F2** = Winning; **F3** = Playboy; **F4** = Violence; **F5** = Heterosexual self-preservation; **F6** = Pursuit of status; **F7** = Primacy of work; **F8** = Power over women; **F9** = Self-reliance; **F10** = Risk-taking.
