# Supplementary material for: Psychometric validation of the Malay CMNI-30: A study among male healthcare professionals in Malaysia
Source: PLoS One. 2025 Apr 1;20(4):e0320765. doi: 10.1371/journal.pone.0320765 (PMC11960922; doi:10.1371/journal.pone.0320765)
Supplement: S4 Table — (DOCX) [file pone.0320765.s005.docx]

**SUPPLEMENTARY DOCUMENT**

S4 Table. Average Variance Extracted (AVE) and Raykov’s rho composite reliability of all ten factors.

| **Code** | **Factor** | **AVE value** | **Raykov’s rho** |
| --- | --- | --- | --- |
| **F1** | Emotional control | 0.511 | 0.689 |
| **F2** | Winning | 0.309 | 0.475 |
| **F3** | Playboy | 0.536 | 0.774 |
| **F4** | Violence | 0.276 | 0.431 |
| **F5** | Heterosexual self-preservation | 0.498 | 0.721 |
| **F6** | Pursuit of status | 0.455 | 0.626 |
| **F7** | Primacy of work | 0.595 | 0.807 |
| **F8** | Power over women | 0.431 | 0.754 |
| **F9** | Self-reliance | 0.192 | 0.270 |
| **F10** | Risk-taking | 0.509 | 0.757 |
