# Supplementary material for: Psychometric validation of the Malay CMNI-30: A study among male healthcare professionals in Malaysia
Source: PLoS One. 2025 Apr 1;20(4):e0320765. doi: 10.1371/journal.pone.0320765 (PMC11960922; doi:10.1371/journal.pone.0320765)
Supplement: S5 Table — (DOCX) [file pone.0320765.s006.docx]

**SUPPLEMENTARY DOCUMENT**

S5 Table. The factor loading Model 9 items and reliability result analysis.

| **Factor** | **Item** | **Factor Loading** | **CI-TC*^a^*** | **Mean inter-item correlation** |
| --- | --- | --- | --- | --- |
| **Emotional control** | X1 | 0.425 | 0.454 | 0.485 |
|  | X2 | 0.668 | 0.620 |  |
|  | X3 | 0.952 | 0.615 |  |
| **Winning** | X4 | 0.495 | 0.302 | 0.302 |
|  | X6 | 0.610 | 0.302 |  |
| **Playboy** | X7 | 0.776 | 0.641 | 0.532 |
|  | X8 | 0.743 | 0.606 |  |
|  | X9 | 0.673 | 0.578 |  |
| **Violence** | X10 | 0.485 | 0.272 | 0.272 |
|  | X11 | 0.562 | 0.272 |  |
| **Heterosexual self-preservation** | X13 | 0.552 | 0.549 | 0.526 |
|  | X14 | 0.794 | 0.700 |  |
|  | X15 | 0.747 | 0.568 |  |
| **Pursuit of status** | X16 | 0.682 | 0.455 | 0.455 |
|  | X18 | 0.667 | 0.455 |  |
| **Primacy of work** | X19 | 0.753 | 0.645 | 0.581 |
|  | X20 | 0.877 | 0.719 |  |
|  | X21 | 0.669 | 0.593 |  |
| **Power over women** | X22 | 0.671 | 0.375 | 0.331 |
|  | X23 | 0.749 | 0.371 |  |
|  | X24 | 0.532 | 0.483 |  |
| **Self-reliance** | X25 | 0.324 | 0.171 | 0.171 |
|  | X26 | 0.529 | 0.171 |  |
| **Risk-taking** | X28 | 0.712 | 0.578 | 0.509 |
|  | X29 | 0.709 | 0.581 |  |
|  | X30 | 0.720 | 0.599 |  |

**Indicator:** *a* = alpha coefficient. **Abbreviations:** CI-TC = corrected item-total correlation.
